# A dual-function host–guest antioxidant system for synergistic treatment of diquat poisoning

**DOI:** 10.1039/d6ra02977a

**Published:** 2026-07-07

**Authors:** Longming Chen, Kaili Jiang, Rongrong Pan, Junhe Ye, Jianou Chen, Yibo Zhao, Tianle Qiu, Pingping Su, Dandan Zhang, Chen Ye, Wenpin Cai, Zhenghao Xu, Xinjun Miao

**Affiliations:** a TCM Science and Research Center, Wenzhou TCM Hospital of Zhejiang Chinese Medical University Wenzhou Zhejiang China wzmiaoxinjun@163.com; b Laboratory of Rheumatology & Institute of TCM Clinical Basic Medicine, College of Basic Medical Science, Zhejiang Chinese Medical University Hangzhou Zhejiang China xuzhenghao@zcmu.edu.cn; c Key Laboratory of Chinese Medicine Rheumatology of Zhejiang Province, College of Basic Medical Science, Zhejiang Chinese Medical University Hangzhou Zhejiang China; d Key Laboratory of Neuropharmacology and Translational Medicine of Zhejiang Province, College of Basic Medical Science, Zhejiang Chinese Medical University Hangzhou Zhejiang China; e College of Chemistry and Molecular Sciences, Henan University Kaifeng Henan Province 475004 China

## Abstract

Diquat (DQ), a widely used herbicide, is highly toxic to humans. DQ ingestion can lead to severe multi-organ dysfunction and high mortality, primarily due to the lack of effective antidotes and detoxification strategies. To address this, we developed a host–guest formulation for the combinatorial treatment of DQ poisoning. This formulation encapsulates ergothioneine (EGT), a potent reactive oxygen species (ROS) scavenger, within the host molecule pillar[6]MaxQ (P6AS). The strong complexation between P6AS and both EGT and DQ was confirmed by NMR and fluorescence titration studies. *In vitro* and *in vivo* evaluations demonstrated the excellent detoxification efficacy of this formulation. The EGT/P6AS complex effectively ameliorated DQ-induced organ damage and promoted the normalization of key hematological and biochemical parameters. Notably, treatment with EGT/P6AS resulted in a 50-percentage-point increase in survival rate, underscoring its remarkable therapeutic potential. These favorable outcomes are attributed to a combinatorial mechanism: DQ triggers the release of EGT to counteract peroxidation damage, while free DQ is simultaneously sequestered within the cavity of P6AS.

## Abstract 2

A supramolecular host–guest formulation based on P6AS and EGT for combinatorial therapy of DQ poisoning.

## Graphical abstract

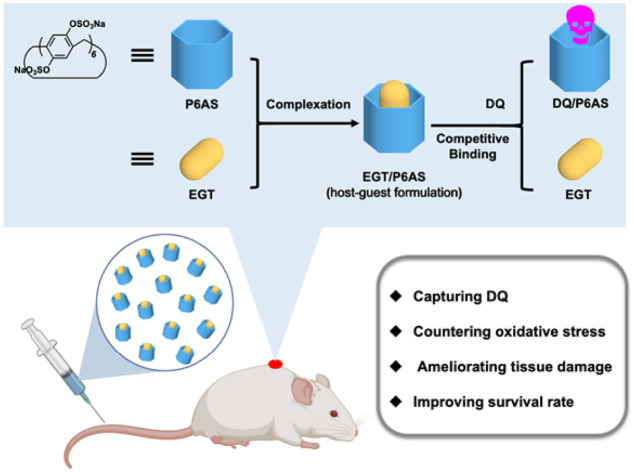

## 1 Introduction

Diquat (DQ), a potent broad-spectrum herbicide, is used in approximately 90% of countries worldwide.^1–4^ Accidental or intentional ingestion of DQ leads to a severe medical condition characterized by life-threatening systemic toxicity and multi-organ failure.^5–8^ In clinical practice, current management strategies focus on accelerating DQ excretion and preventing further absorption, employing methods such as forced diuresis, blood purification, and general supportive care, *etc.*^9,10^ However, these interventions often fail in severe cases, and the lack of a targeted antidote continues to contribute to high death rates.^1,10,11^ Besides, although the toxicity of DQ is primarily attributed to excessive generation of reactive oxygen species (ROS), such as H_2_O_2_, ˙O_2_^−^ and HO˙ *via* redox cycling, existing antioxidant therapies are largely nonspecific and inadequately effective.^12–14^ Thus, managing DQ intoxication continues to pose a significant clinical challenge.

Given these limitations of existing DQ intoxication therapies, we propose a cooperative host–guest supramolecular strategy. This system employs a water-soluble macrocyclic host that can encapsulate antioxidant guests through reversible noncovalent interactions. By forming host–guest complexes, the macrocycle sequesters circulating and tissue-accumulated DQ to promote its clearance, while the co-delivered antioxidant is responsively released to mitigate oxidative damage, thereby achieving a synergistic therapeutic effect.

**Scheme 1:**
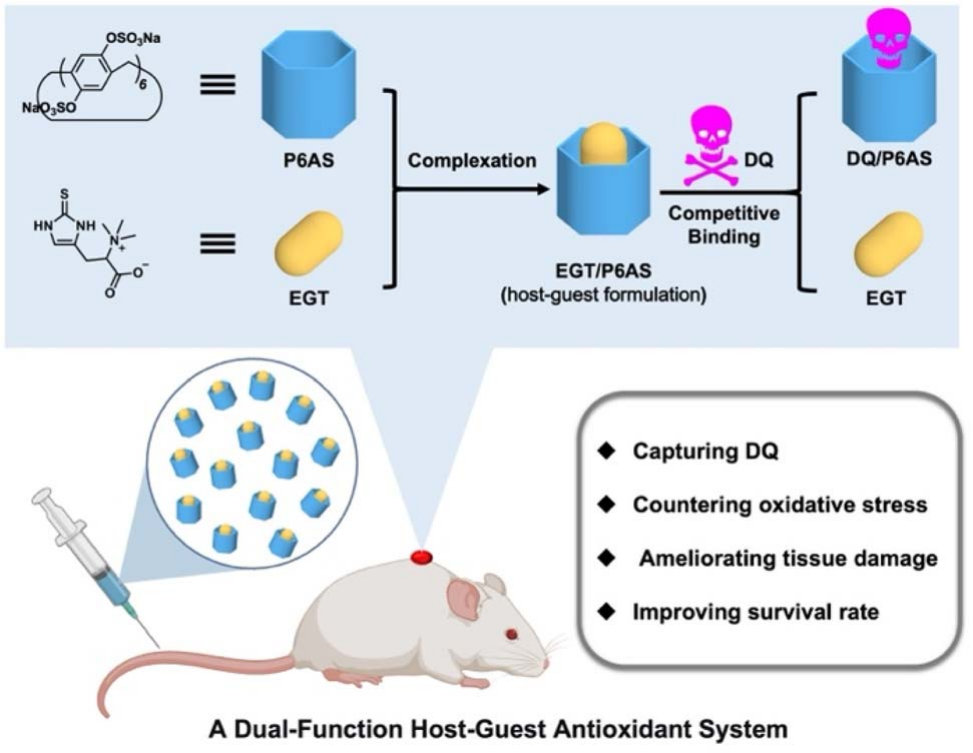
Schematic illustration of the designed principle of dual-function host–guest antioxidant system and its mechanism of synergistic treatment of DQ poisoning.

As a proof of concept, we constructed a host–guest formulation based on pillar[6]MaxQ (P6AS) and ergothioneine (EGT). EGT is a potent and physiologically stable ROS scavenger suitable for countering DQ-induced oxidative stress.^15–18^ P6AS, a widely used water-soluble pillararene derivative, was selected as the macrocyclic host due to its excellent biocompatibility and favourable size/charge complementarity with both EGT and DQ.^19–23^ In this formulation, DQ triggers the release of EGT to mitigate peroxidation damage, while concurrently being encapsulated within the P6AS cavity. This dual mechanism enables a combinatorial therapy aimed at improving survival and organ recovery in DQ poisoning (Scheme 1).

## 2 Results and discussion

### 2.1 Host–guest complexation studies

**Fig. 1:**
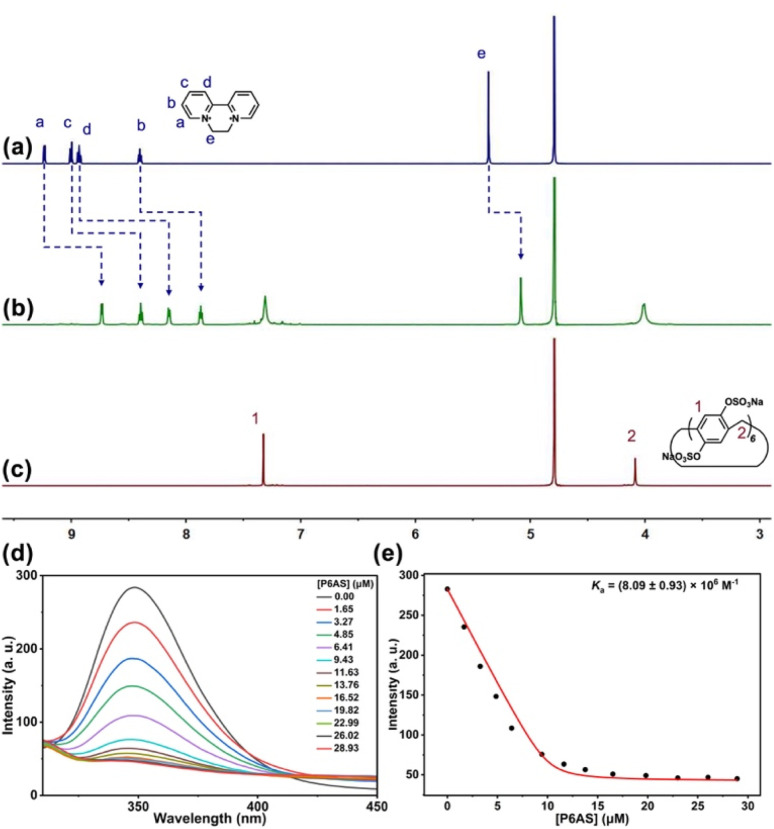
Host–guest recognition studies. ^1^H NMR spectra (600 MHz, D_2_O) of (a) DQ (5.00 mM), (b) DQ (5.00 mM) with addition of P6AS (5.00 mM) and (c) P6AS (5.00 mM). (d) Direct fluorescence titration of DQ (10.0 µM) with P6AS in aqueous solution, *λ*_ex_ = 275 nm. (e) The associated titration curve at *λ*_em_ = 350 nm and fitted according a 1 : 1 binding stoichiometry.

The design principle of our supramolecular formulation is based on the ability of P6AS to bind EGT and release it in response to DQ. To confirm this, we first investigated the host–guest complexation between P6AS and the two guests (EGT and DQ) using ^1^H NMR spectroscopy. As shown in Fig. 1a–c, the NMR spectrum of DQ in D_2_O was recorded in the absence and presence of P6AS. Upon addition of one equivalent of P6AS, all proton signals of DQ exhibited considerable upfield shifts and broadening compared to free DQ, presumably as a consequence of the inclusion-induced shielding effect. The proton signals H_1_ and H_2_ of P6AS also shifted upfield, likely due to electrostatic interactions with the guest. A similar complexation behaviour was observed between EGT and P6AS (Fig. S2). To further support these findings, geometry optimization of the DQ/P6AS and EGT/P6AS complexes was performed using AutoDock Vina. The resulting models confirmed the formation of 1 : 1 complex, with both guests positioned inside the cavity of P6AS (Fig. S3). Collectively, these results demonstrate that P6AS can form stable complexes with both EGT and DQ at room temperature.

We next quantified the binding affinities of P6AS with EGT and DQ using fluorescence titration.^24–32^ Job's plot analysis (continuous variation method) and isothermal titration calorimetry (ITC) confirmed a 1 : 1 binding stoichiometry for both systems (Fig. S4 and S5). Direct fluorescence titration was then performed to determine the association constants (*K*_a_) in aqueous solution. As shown in Fig. 1d, the fluorescence intensity of P6AS gradually decreased upon incremental addition of DQ, which were attribute to photo-induced electron transfer.^30–32^ The *K*_a_ value for DQ/P6AS was determined to be (8.09 ± 0.93) × 10^6^ M^−1^ by a standard curve fitting protocol (Fig. 1e). In comparison, the *K*_a_ for EGT/P6AS was significantly lower [(3.42 ± 0.02) × 10^4^ M^−1^, Fig. S6]. This substantial difference in binding affinity provides a thermodynamic basis for the competitive displacement of EGT by DQ from the EGT/P6AS complex, enabling the proposed triggered-release mechanism. Finally, the binding selectivity of P6AS toward DQ was evaluated in the presence of potential biological interferents. As shown in Fig. S7, common endogenous substances, including neurotransmitters, amino acids, metal ions and serum proteins, caused no significant change in the fluorescence of the DQ/P6AS system, confirming the high selectivity of P6AS for DQ in complex media.

### 2.2 Safety profile of P6AS

**Fig. 2:**
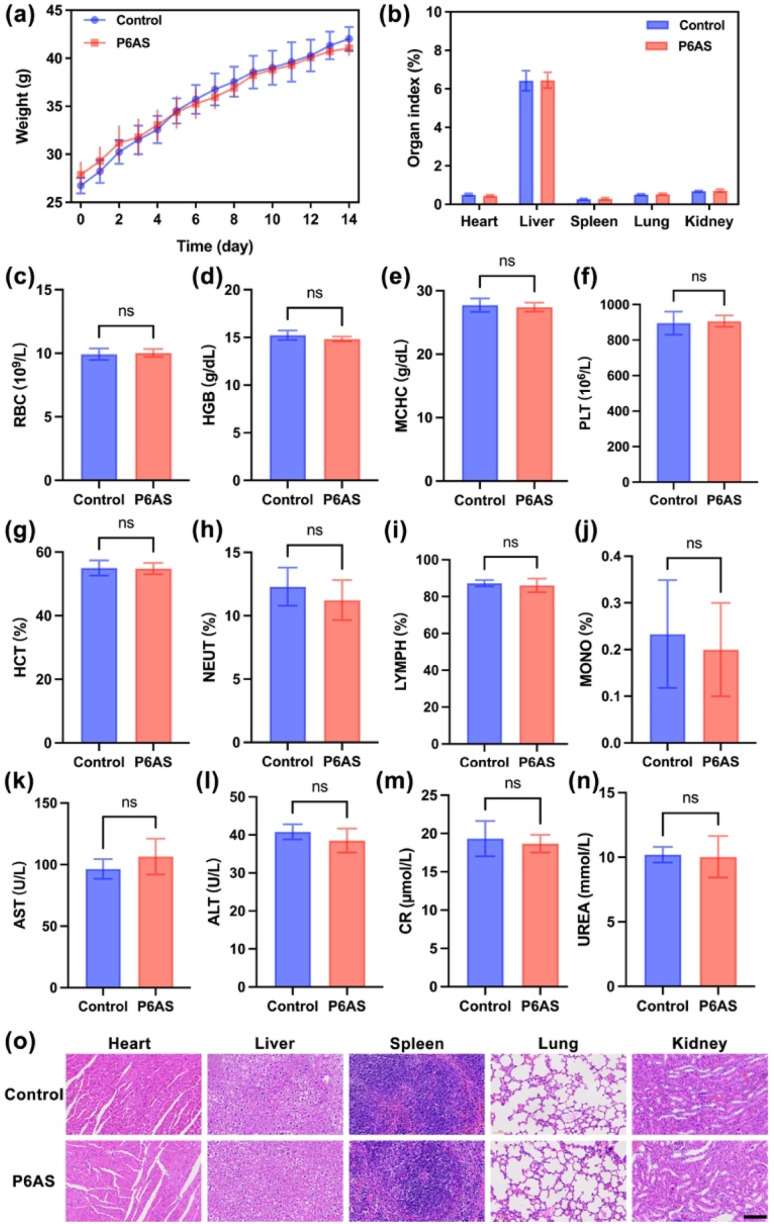
Safety profile of P6AS. (a) Change in body weight of mice administrated with P6AS (100 mg kg^−1^). (b) Major organ indexes of the mice on day 14 post-administration of P6AS. (c) RBC, (d) HGB, (e) MCHC, (f) PLT, (g) HCT%, (h) NEUT%, (i) LYMPH%, (j) MONO%, (k) AST, (l) ALT, (m) CR and (n) UREA levels at day 14 after treatment with P6AS. (o) H&E stains of the major organs from the mice injected with P6AS. The scale bar is 100 µm. P6AS: pillar[6]MaxQ; RBC: red blood cells; HGB: hemoglobin; MCHC: mean corpuscular hemoglobin concentration; PLT: platelets; HCT: hematocrit; NEUT: neutrophils; LYMPH: lymphocytes; MONO: monocytes; AST: aspartate transaminase; ALT: alanine transaminase; CR: creatinine. Statistical significance was assessed by *t*-test; ns, no significance.

Prior to biological activity analysis, we thoroughly assessed the safety profile of P6AS. Cytotoxicity was first examined *in vitro* using human hepatocellular carcinoma (HepG2), normal renal epithelial (293T), and non-small-cell lung cancer (A549) cell lines *via* a standard CCK 8 assay. As shown in Fig. S8–S10, P6AS exhibited minimal cytotoxicity across all cell lines, even at relatively high concentrations. The systemic biocompatibility of P6AS was then evaluated in Kunming mice following intravenous injection (100 mg kg^−1^). Body weight was monitored over a two-period. As shown in Fig. 2a, weight fluctuations in the P6AS-treated group were comparable to those in the PBS control group. On day 14 post-administration, all mice were euthanized, and major organs (heart, liver, spleen, lungs, kidneys) together with blood samples were collected for further analysis. Organ indexes showed no significant differences between the P6AS and PBS groups (Fig. 2b). Comprehensive hematological and biochemical analyses including red blood cells (RBC), hemoglobin (HGB), mean corpuscular hemoglobin concentration (MCHC), platelets (PLT), hematocrit (HCT), neutrophils (NEUT), lymphocytes (LYMPH), monocytes (MONO), aspartate transaminase (AST), alanine transaminase (ALT), creatinine (CR), and urea (Fig. 2c–n) revealed no significant alterations in blood parameters, hepatic function, and renal function following P6AS administration. Further evidence of biocompatibility was provided by histopathological examination of major organs (Fig. 2o). Compared with the PBS group, tissues from P6AS-treated mice displayed no detectable inflammatory infiltration or toxicological damage. Collectively, these *in vitro* and *in vivo* safety assessments indicated that P6AS possesses an excellent biocompatibility profile, supporting its suitability for subsequent biological efficacy studies. We further evaluated the storage stability of the EGT/P6AS complex, which is critical for its potential translation and practical application. The fluorescence intensity of the complex was monitored over time under two representative storage conditions (4 °C and 25 °C). Samples were analyzed at 1, 3, and 7 days after preparation. As shown in Fig. S11, no significant change in fluorescence emission was observed under either condition throughout the 7 days observation period. These results indicated that the EGT/P6AS complex remained intact and stable during short-term storage at both refrigerated and ambient temperatures.

### 2.3 Treatment of DQ poisoning by EGT/P6AS *in vitro*

**Fig. 3:**
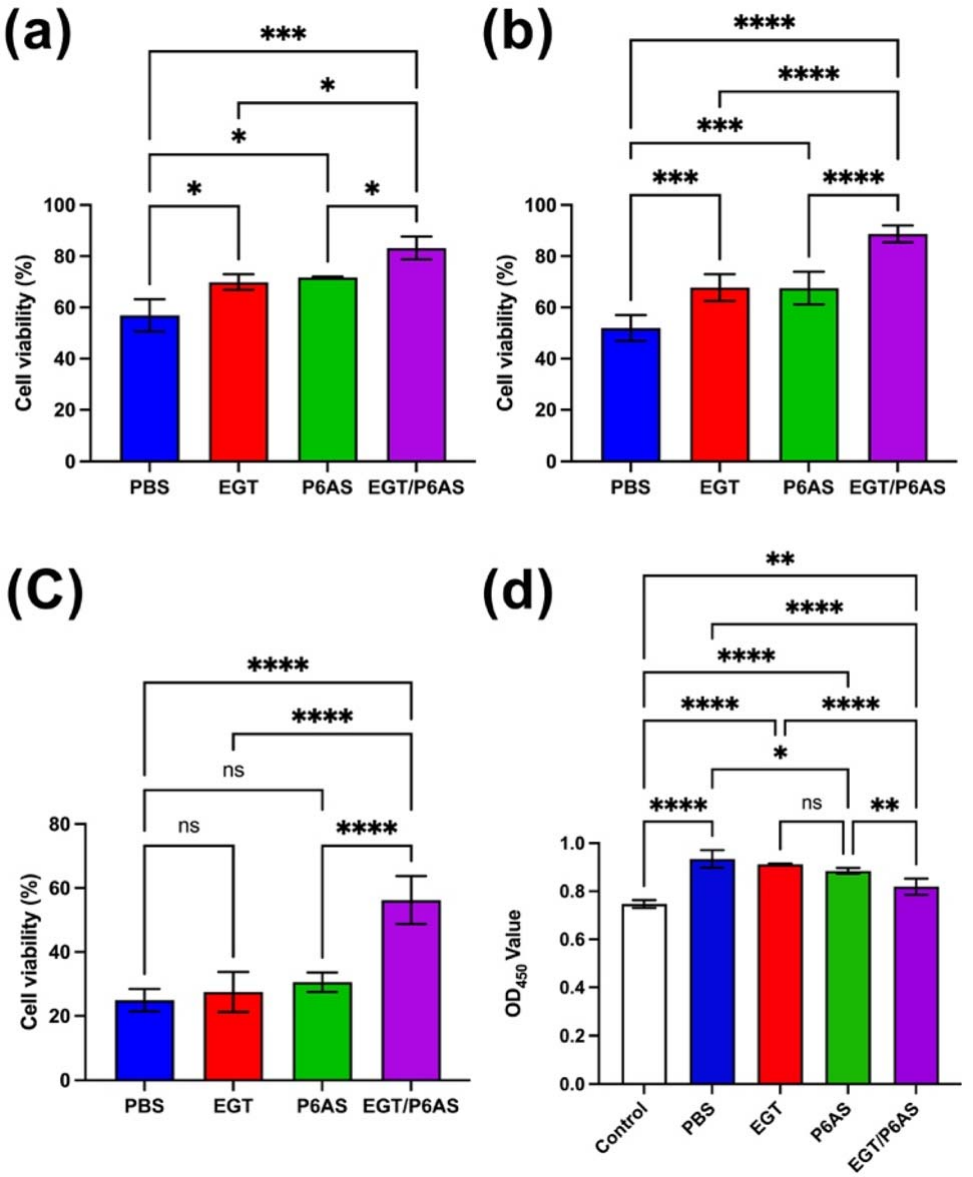
*In vitro* detoxification efficacy of different formulations against DQ poisoning. Cell viability of (a) HepG2, (b) 293T, and (c) A549 cells after 24 h treatment with DQ (100 µM) in the presence of PBS, EGT, P6AS, or EGT/P6AS. (d) LDH release from 293T cells treated under the same conditions. Data are presented as mean ± SD (*n* = 5). *P*-values are determined using one-way analysis of variance (ANOVA) test. ns, no significance. *P < 0.05, **P < 0.01, ***P < 0.001, and ****P < 0.0001.

Based on the strong binding affinity of P6AS toward DQ and its excellent biocompatibility, we next systematically evaluated the protective efficacy of this formulation against DQ-induced cytotoxicity *in vitro*. As shown in Fig. S12–S14, DQ exhibited a concentration-dependent cytotoxic effect across HepG2, 293T, and A549 cell lines within 24 h of exposure. Co-treatment with either P6AS or EGT (1 equivalent) significantly attenuated the cytotoxicity induced by 100 µM DQ. Notably, the EGT/P6AS formulation demonstrated the most pronounced cytoprotective activity. In HepG2 cells exposed to 100 µM DQ, from (56.92 ± 5.15)% in the PBS group to (83.24 ± 3.64)% with EGT/P6AS treatment, significantly higher than that achieved with P6AS alone [(71.70 ± 0.31)%] or free EGT [(69.90 ± 2.48)%] (*P* < 0.05 for EGT/P6AS group *vs.* P6AS group; *P* < 0.05 for EGT/P6AS group *vs.* EGT group; Fig. 3a). A consistent protective trend was also observed in both 293T (*P* < 0.001 for EGT/P6AS group *vs.* P6AS group; *P* < 0.001 for EGT/P6AS group *vs.* EGT group; Fig. 3b) and A549 cell lines (*P* < 0.001 for EGT/P6AS group *vs.* P6AS group; *P* < 0.001 for EGT/P6AS group *vs.* EGT group; Fig. 3c), confirming the broad-spectrum efficacy of the host–guest formulation. Additionally, a similar protective efficacy trend was observed upon exposure to 200 µM DQ in three cell lines, indicating that the formulation remains effective across a broader dose range (Fig. S15). To further evaluate membrane integrity, lactate dehydrogenase (LDH) release was quantified. DQ (100 µM) exposure led to a significant increase in extracellular LDH, indicative of membrane damage (Fig. 3d). This effect was markedly suppressed by treatment with P6AS, EGT, or the EGT/P6AS complex, with the latter exhibiting the strongest membrane-stabilizing effect.

Given the central role of oxidative stress in DQ toxicity, intracellular ROS levels were examined in 293T cells using fluorescence microscopy. DQ treatment induced substantial ROS accumulation, which was effectively mitigated by both EGT and P6AS alone (Fig. S16). Importantly, the EGT/P6AS combination elicited the most significant reduction in ROS levels, underscoring its superior antioxidant capacity *in vitro*. Taken together, these findings demonstrate that the host–guest formulation of EGT with P6AS confers a synergistic detoxification effect against DQ poisoning in cellular models, primarily through enhanced membrane protection and ROS scavenging.

### 2.4 Therapeutic effects of EGT/P6AS for DQ poisoning *in vivo*

**Fig. 4:**
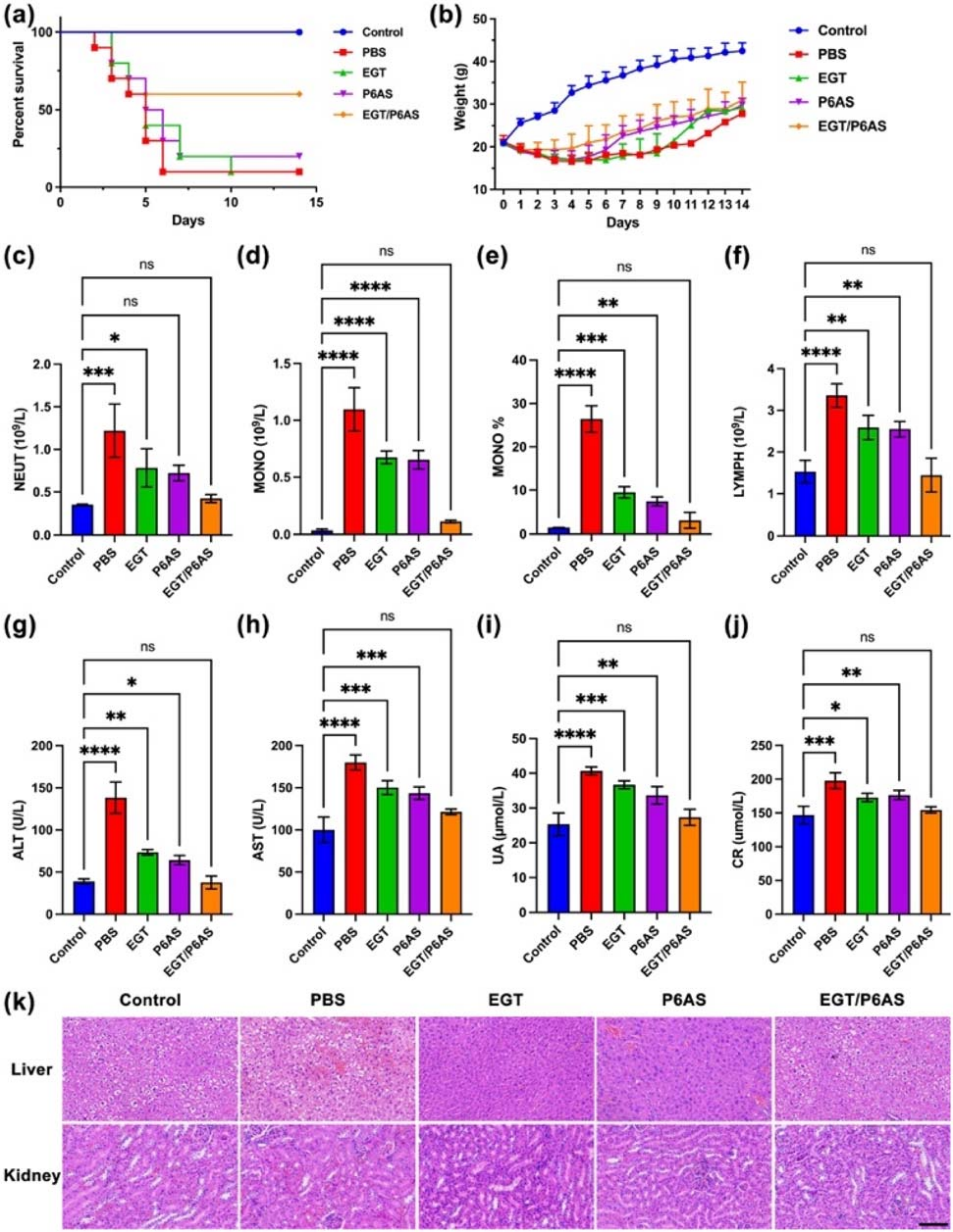
Therapeutic effects of EGT/P6AS for DQ poisoning *in vivo*. (a) Kaplan–Meier survival curves of DQ poisoning mice in several group (*n* = 10). (b) Change in body weight of DQ poisoning mice in several group (*n* = 10). (c) NEUT, (d) MONO, (e) MONO%, (f) LYMPH, (g) ALT, (h) AST, (i) UA, and (j) CR levels determined at day 2 after mice had administrated with DQ (60 mg kg^−1^) by intraperitoneal injection and treatment with different formulations. (k) H&E stains of the kidney and liver from the mice in different treatment groups after DQ poisoning. Untreated normal mice served as control group. The scale bar is 100 µm. *P*-values are determined using one-way analysis of variance (ANOVA) test. ns, no significance. *P < 0.05, **P < 0.01, ***P < 0.001, and ****P < 0.0001.

Encouraged by the promising *in vitro* results, we further evaluated the therapeutic efficacy of EGT/P6AS for DQ poisoning in mice. Kunming mice (body weight ∼20 g) received an intraperitoneal injection of a high dose of DQ (60 mg kg^−1^) and were subsequently treated with PBS, P6AS (54 mg kg^−1^), free EGT (7.6 mg kg^−1^, equimolar to P6AS), or the EGT/P6AS complex [54 mg kg^−1^ P6AS + 7.6 mg kg^−1^ EGT] at predetermined intervals. As shown in Fig. 4a and b, the PBS-treated group exhibited a mortality rate of 90% by day 6, accompanied by a sharp decline in body weight. The survival curve of the free EGT group was comparable to that of the PBS group. Treatment with P6AS alone increased the survival rate to 20%. Notably, administration of the EGT/P6AS complex significantly improved outcomes, achieving a 60% survival rate. Surviving mice in this group also displayed stable body weight trajectories, with weight recovery beginning by day 3 post-exposure.

To assess systemic toxicity, a subset of mice was euthanized on day 2 for hematological and biochemical analyses. DQ intoxication markedly elevated several inflammatory markers, including neutrophil (NEUT, *p* < 0.001), monocyte (MONO, *p* < 0.0001), monocyte percentage (MONO%, *p* < 0.0001), and lymphocyte (LYMPH, *p* < 0.0001) counts (Fig. 4c–f), indicating a pronounced systemic inflammatory response. Serum biochemical parameters also revealed significant hepatic and renal injury, with sharp increases in ALT (*p* < 0.0001), AST (*p* < 0.0001), uric acid (UA, *p* < 0.001), and creatinine (CR, *p* < 0.0001) levels (Fig. 4g–j), consistent with prior reports. These abnormalities were partially attenuated by treatment with either P6AS or free EGT, with the most pronounced normalization observed in the EGT/P6AS group. As shown in Fig. 4k, histopathological examination of liver tissue revealed portal inflammation and scattered hepatocyte necrosis in DQ-exposed mice, consistent with severe hepatic injury. These pathological changes were moderately alleviated by free EGT or P6AS alone, while the EGT/P6AS complex exhibited the greatest protective effect, with minimal observable histopathological damage. Similarly, renal tissues from DQ-treated mice showed tubular dilation, atrophy, interstitial expansion, and inflammatory infiltration, all of which were markedly reduced by P6AS or EGT treatment, and most effectively suppressed by the EGT/P6AS formulation. Collectively, these *in vivo* results demonstrated that the host–guest complex of EGT and P6AS exerts a synergistic therapeutic effect against DQ poisoning, significantly improving survival, attenuating systemic inflammation, and mitigating organ-specific damage.

## 3 Conclusion

In summary, this study successfully developed and validated a supramolecular host–guest formulation based on P6AS and EGT as a novel antidote for combinatorial therapy of DQ poisoning. The rationally designed system leverages the strong and selective binding affinity of P6AS for DQ, coupled with the potent antioxidant activity of EGT, to establish a DQ-triggered release mechanism. Comprehensive *in vitro* assessments confirmed the superior cytoprotective efficacy of the EGT/P6AS complex, evidenced by enhanced cell viability, reduced LDH release, and significant attenuation of intracellular ROS levels. The therapeutic potential of this strategy was unequivocally demonstrated in a murine model of acute DQ poisoning. Treatment with the EGT/P6AS formulation resulted in a substantial survival benefit, alongside the amelioration of systemic inflammation and the normalization of key hematological and biochemical markers indicative of hepatic and renal function. Histopathological analysis further corroborated the robust protective effects, showing minimal organ damage compared to severe injury in the PBS group. Collectively, our findings establish that the synergistic action of this supramolecular system, simultaneously sequestering circulating DQ and releasing its antioxidant cargo in response to the toxin, offers a highly effective and mechanistically coherent strategy for treating DQ intoxication. This work not only presents a promising therapeutic candidate but also provides a conceptual framework for designing stimulus-responsive detoxification platforms against other toxic agents.

## Ethical statement

All animal procedures were approved by the Wenzhou Institute of the Chinese Academy of Sciences and conducted in accordance with the guidelines of the Institutional Animal Care and Use Committee (IACUC). All experimental protocols complied with the ethical standards set forth by the Association for Assessment and Accreditation of Laboratory Animal Care International (AAALAC).

## Conflicts of interest

There are no conflicts to declare.

## Supplementary Material

RA-OLF-D6RA02977A-s001

## Data Availability

The data supporting this article have been included as part of the supplementary information (SI). Supplementary information is available. See DOI: https://doi.org/10.1039/d6ra02977a.
