# Supplementary material for: A dual-function host–guest antioxidant system for synergistic treatment of diquat poisoning
Source: RSC Adv. 2026 Jul 7;16(36):38449–54. doi: 10.1039/d6ra02977a (PMC13339991; doi:10.1039/d6ra02977a)
Supplement: Supplementary file 1 [file RA-016-D6RA02977A-s001.pdf]

Supplementary Information (SI) for RSC Advances.
This journal is © The Royal Society of Chemistry 2026

Supporting Information

A Dual-Function Host-Guest Antioxidant System for Synergistic Treatment of Diquat Poisoning

Longming Chen,‡[a] Kaili Jiang,‡[a] Rongrong Pana,‡[a] Junhe Y,[b] Jianou Chen,[a] Yibo Zhao,[c] Tianle Qiu,[a] Pingping Su,[a] Dandan Zhang,[a] Chen Ye,[a] Wenpin Cai,*[a] Zhenghao Xua,*[b] and Xinjun Miao*[a]

[a] *TCM Science and Research Center, Wenzhou TCM Hospital of Zhejiang Chinese Medical University, Wenzhou, Zhejiang, China.*

[b] *Laboratory of Rheumatology & Institute of TCM Clinical Basic Medicine, College of Basic Medical Science, Zhejiang Chinese Medical University, Hangzhou, Zhejiang, China; Key Laboratory of Chinese Medicine Rheumatology of Zhejiang Province, College of Basic Medical Science, Zhejiang Chinese Medical University, Hangzhou, Zhejiang, China; Key Laboratory of Neuropharmacology and Translational Medicine of Zhejiang Province, College of Basic Medical Science, Zhejiang Chinese Medical University, Hangzhou, Zhejiang, China.*

[c] *College of Chemistry and Molecular Sciences, Henan University, Kaifeng, Henan, China.*

[1] These authors contributed to the work equally.

Table of Contents

1 General materials and methods S2

1.1 Materials S2

1.2 Cells and Animals

1.3 Instruments S2

1.4 Fluorescence titration S2

1.5 Cytotoxicity assay S3

1.6 Determination of ROS level *in vitro* S3

1.7 Treatment protocols S3

2 Synthesis of P6AS S4

2.1 Synthesis of P6AS S4

3 Supporting results and experimental raw data S5

3.1 Characterization of P6AS S5

3.2 ^{1}H NMR of EGT and P6AS S5

3.3 Optimized geometries of guests (DQ and EGT) with P6AS S6

3.4 Job's plot analysis and ITC for complexation of guests (DQ and EGT) with P6AS S7

3.5 Binding of P6AS with EGT S8

3.6 Binding of P6AS with biological species S8

3.7 Biosafety evaluation of P6AS S9

3.8 The storage stability of the EGT/P6AS complex S10

3.9 Treatment of DQ poisoning by EGT/P6AS *in vitro* S11

3.10 Intracellular ROS levels in 293T cells S12

References S13

1 General materials and methods

1.1 Materials. All reagents were obtained from commercial suppliers and used as received unless otherwise specified. Diquat (DQ) and ergothioneine (EGT) were purchased from Energy Chemical. Dimethyl sulfoxide (DMSO), methanol, acetonitrile, Dulbecco's modified Eagle's medium (DMEM), penicillin-streptomycin, and phosphate-buffered saline (PBS) were obtained from Thermo Fisher Scientific. Fetal bovine serum (FBS) was purchased from Excell. The Cell Counting Kit-8 (CCK-8) was obtained from Beyotime. Human hepatocellular carcinoma (HepG2), human normal renal epithelial (293T), and human non-small cell lung cancer (A549) cell lines were procured from the Cell Bank of the Chinese Academy of Sciences. Pillar[6]MaxQ (P6AS) was synthesized and purified following previously reported procedures.[1-2]

1.2 Cells and Animals. HepG2, 293T and A549 cells were cultured in DMEM supplemented with 10% FBS, 1% penicillin and 1% streptomycin. Then cells were incubated at 37 °C under 5% CO_2 and 90% relative humidity, and passaged every 2 days.

KM mice with 18-22 g weight were purchased from the SPF Biotechnology Co. Ltd. All animal procedures were approved by the Wenzhou Institute of the Chinese Academy of Sciences and conducted in accordance with the guidelines of the Institutional Animal Care and Use Committee (IACUC). Mice were housed in groups of five per cage under controlled environmental conditions: temperature (22 ± 2) °C and a 12 h light/12 h dark cycle. All experimental protocols complied with the ethical standards set forth by the Association for Assessment and Accreditation of Laboratory Animal Care International (AAALAC).

1.3 Instruments. ^{1}H NMR spectrums were recorded using a Bruker AVANCE 600 MHz spectrometer. Fluorescence spectroscopic studies were carried out using a F97-pro fluorescence spectrophotometer, Lengguang Tech Co. Ltd. Cytotoxicity and LDH studies were performed on SpectraMax ® Cmax Plus plate reader, Molecular Devices.

1.4 Fluorescence titration. Fluorescence titrations of host (P6AS) with guests (EGT and DQ) were carried out to qualitatively determine their association constants (K_a) in aqueous solution. The complexation of the host and the gust was measured according to the procedure reported previously[3-5].

1.5 Cytotoxicity assay. The cells (HepG2, 293T and A549) were seeded in 96-well plates at a density of 10^4 cells per well and cultured for 24 h. Then cells were incubated with DQ (100 μM) in the presence or absence of P6AS (100 μM), EGT (100 μM) or EGT/P6AS (100/100 μM) for 24h. The cell viability of HepG2, 293T and A549 was determined using a Cell Counting Kit-8 (CCK-8) assay.

1.6 Determination of ROS level *in vitro*. The fluorescence probe (2’,7’-dichlorodihydrofluorescein diacetate) DCFH-DA can be oxidized to the highly fluorescent dichlorofluorescein (DCF) by intracellular ROS. ROS level was determined using a fluorescence microscope. Briefly, 293T cells were plated into a 6-well culture plates at a density of 10^5 cells per well and incubated for 24 h. Then the cells were treated with DQ (100 μM) and different formulations [P6AS (100 μM), EGT (100 μM) or EGT/P6AS (100/100 μM)] for 24h. The medium was replaced with PBS stained with Hoechst and DCFH-DA (10μM) for 30 min according to the product instructions. Then the ROS level was determined using a fluorescence microscope.

1.7 Treatment protocols. Male KM mice with weights of 18-22 g were administered with DQ at a dose of 60 mg/kg by intraperitoneal injections. After administration of DQ, each group of mice was administered 5 consecutive times of injection of PBS, P6AS (54 mg/kg), EGT (7.6 mg/kg, the equal equivalent of P6AS) or EGT/P6AS [P6AS (54 mg/kg) + EGT (7.6 mg/kg)] *via* the intravenous route at 6 h, 12 h, 24 h, 48 h, and 72 h, respectively.

2 Synthesis of P6AS.

2.1 Synthesis of P6AS

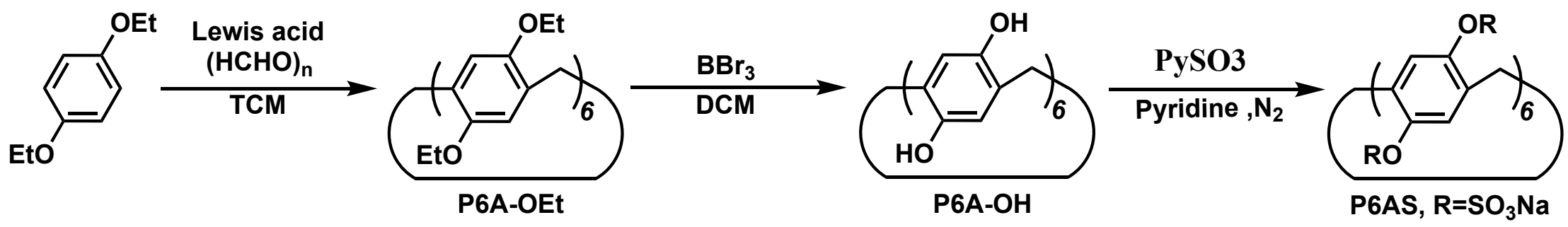

Scheme S1. Synthetic route of P6AS.

P6AS were synthesized and purified according to the procedure reported previously.[1,2]

3 Supporting results and experimental raw data

3.1 Characterization of P6AS.

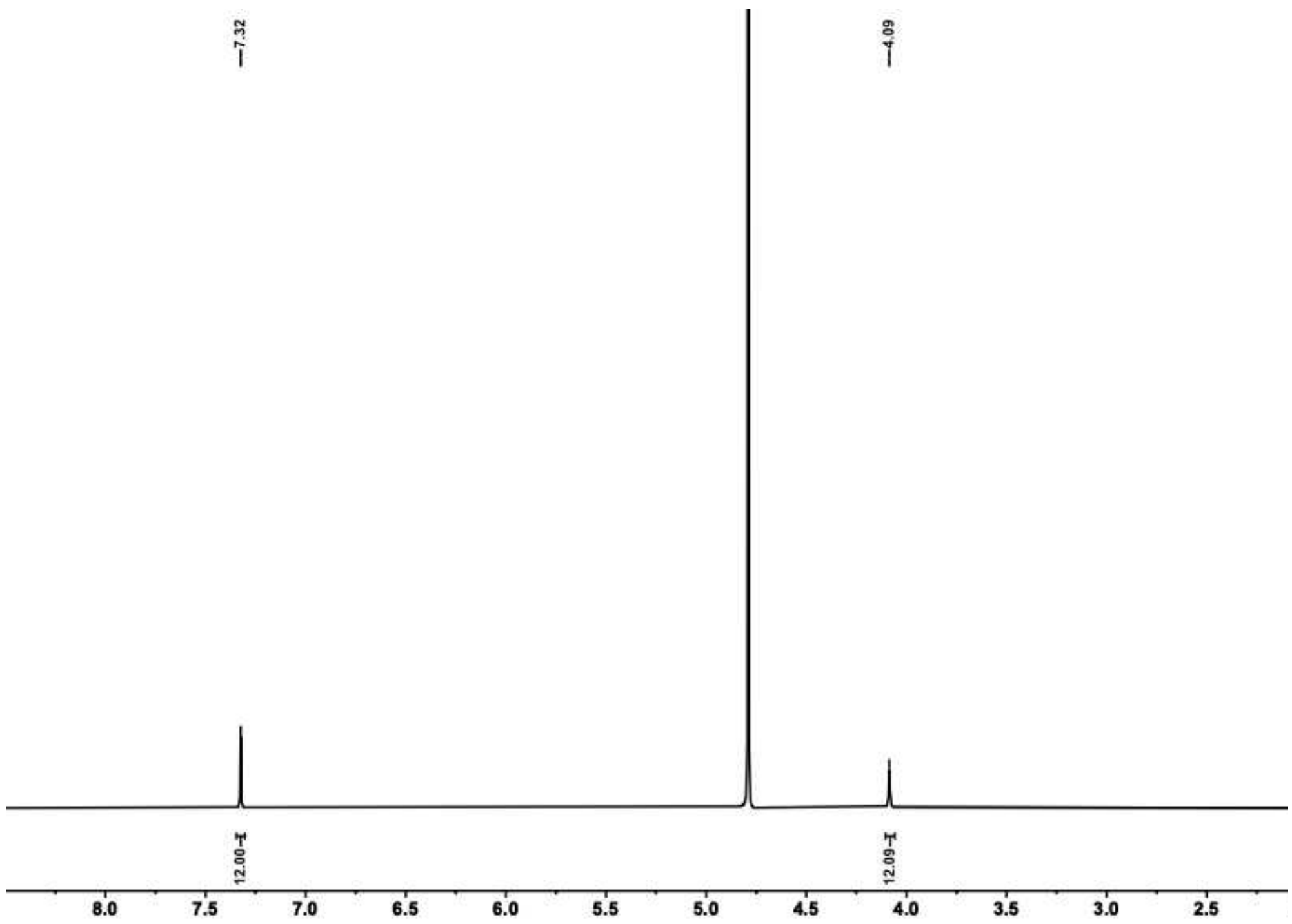

Fig. S1. ^{1}H NMR spectrum of P6AS in D_2O, 600 MHz.

3.2 ^{1}H NMR of EGT and P6AS.

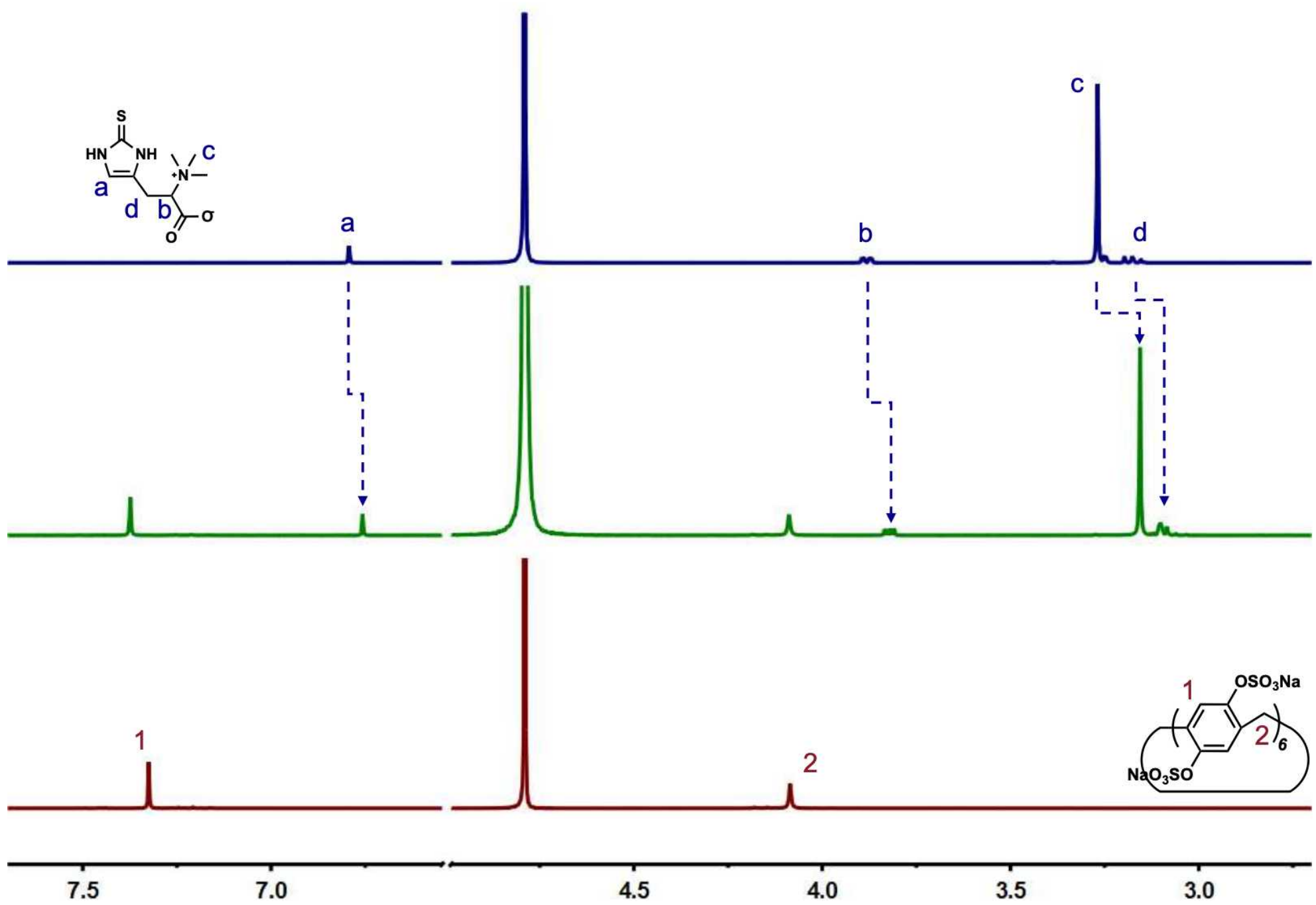

Fig. S2. ^{1}H NMR spectra (600 MHz, D_2O) of (a) EGT (5.00 mM), (b) EGT (5.00 mM) with addition of P6AS (5.00 mM) and (c) P6AS (5.00 mM).

3.3 Optimized geometries of guests (DQ and EGT) with P6AS.

(a) (b)

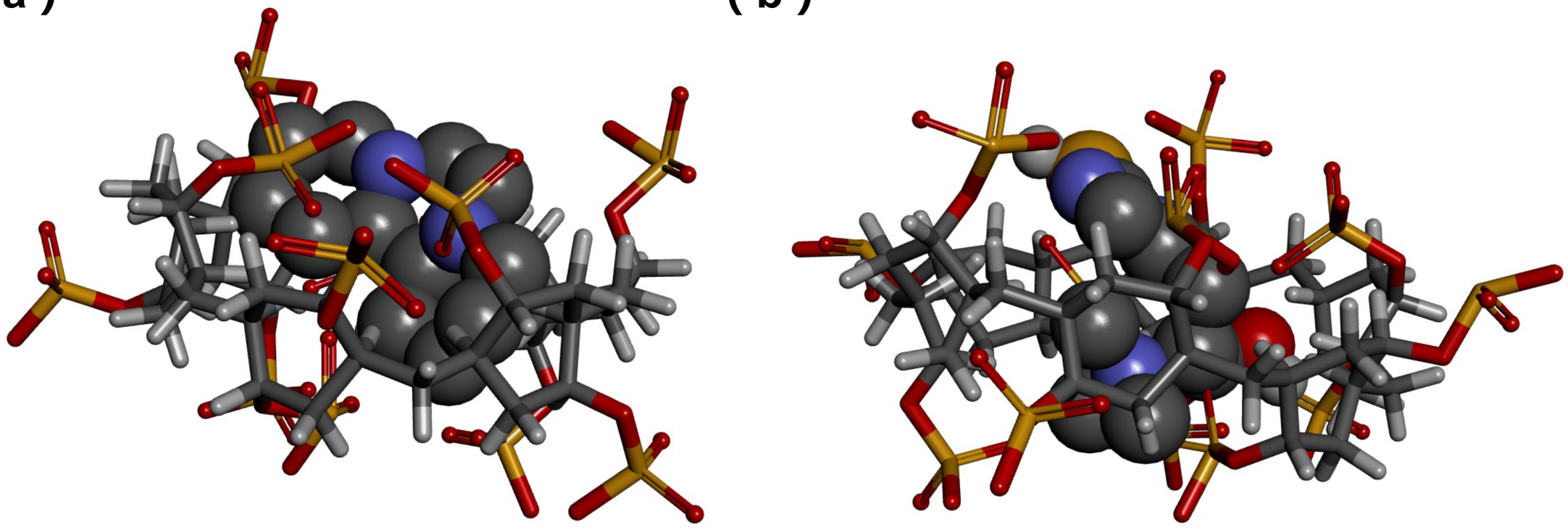

Fig. S3. Molecular docking simulation of (a) DQ/P6AS and (b) EGT/P6AS.

3.4 Job's plot analysis and ITCfor complexation of guests (DQ and EGT) with P6AS.

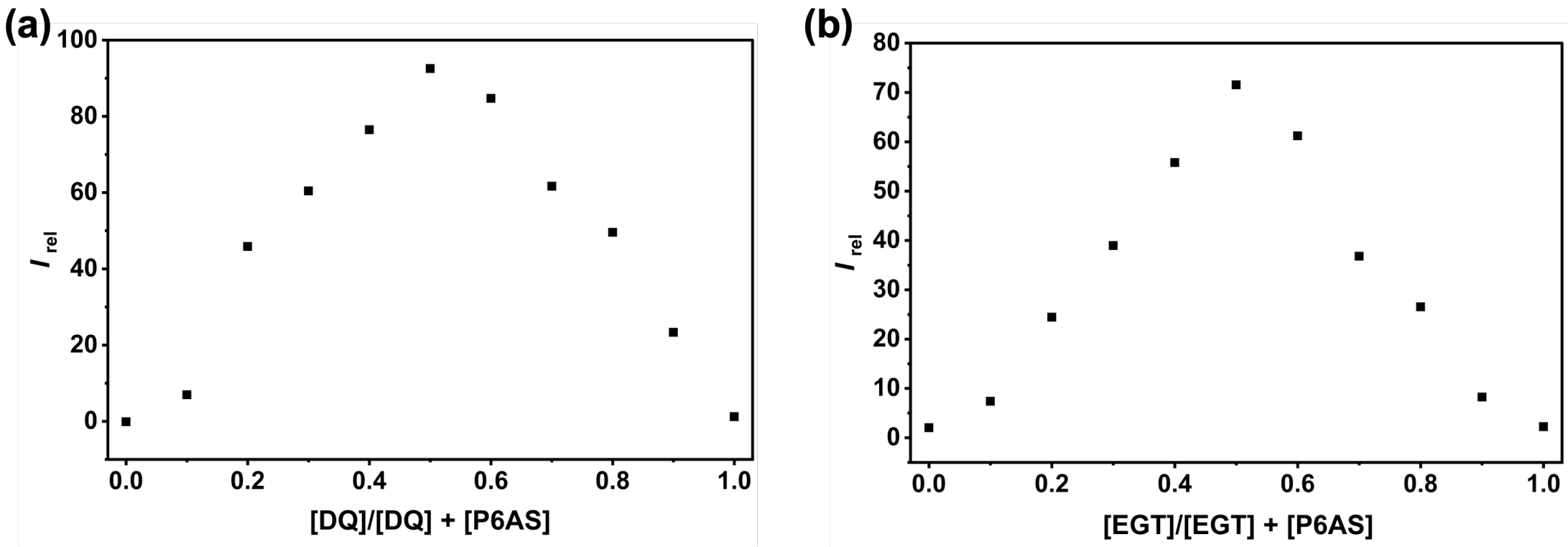

Fig. S4. Job's plot for P6AS with (a) DQ and (b) EGT in aqueous solution at room temperature (λ_{ex} = 275 nm, [DQ] + [P6AS] = 10 μM; λ_{ex} = 290 nm, [P6AS] + [EGT] = 10 μM).

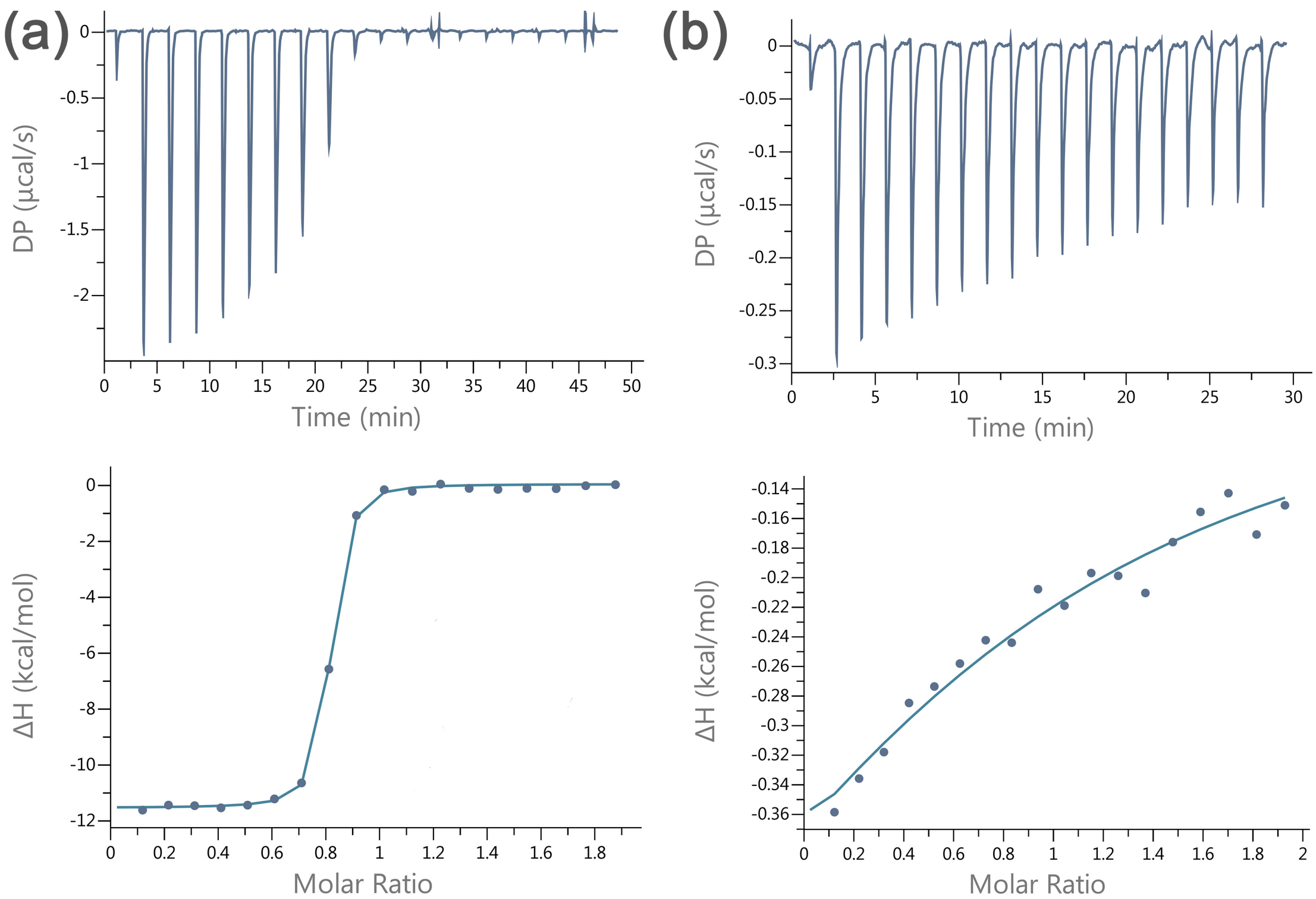

Fig. S5. ITC titration of DQ/P6AS and EGT/P6AS in 10 mM PBS buffer at 298 K. One binding site model was utilized to fit the data.

3.5 Binding of P6AS with EGT.

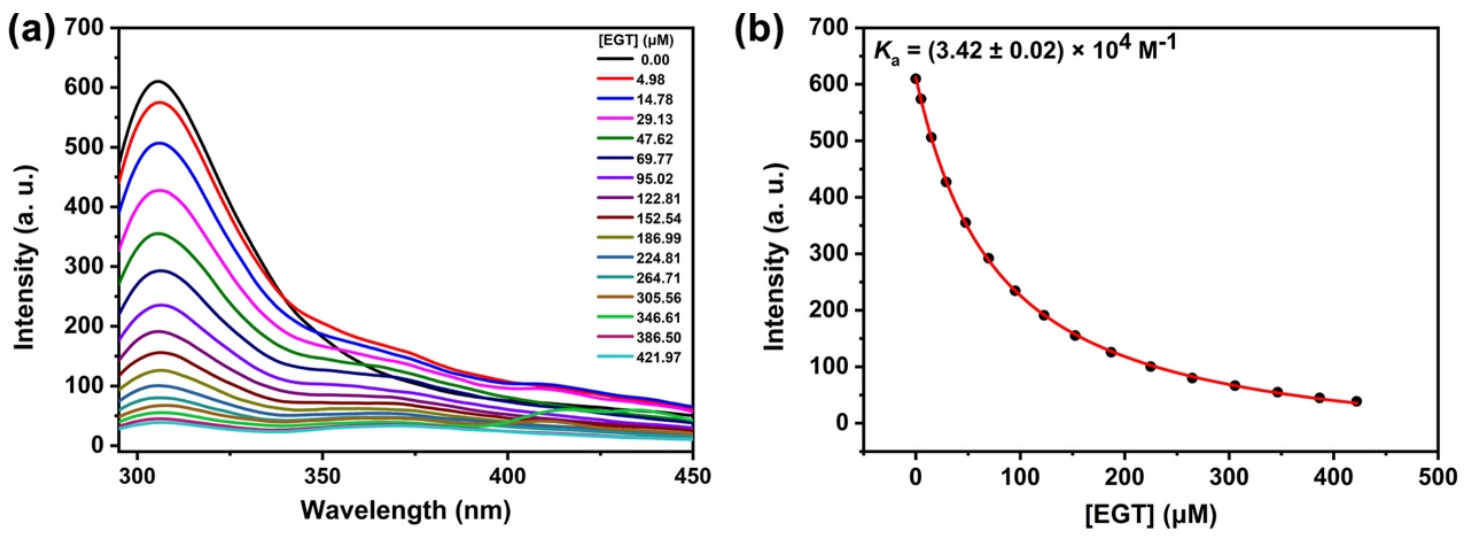

Fig. S6. (a) Direct fluorescence titration of P6AS (10 μM) with EGT in aqueous solution, λ_{ex} = 290 nm. (b) The associated titration curve at λ_{em} = 310 nm and fitted according a 1:1 binding stoichiometry.

3.6 Binding of P6AS with biological species.

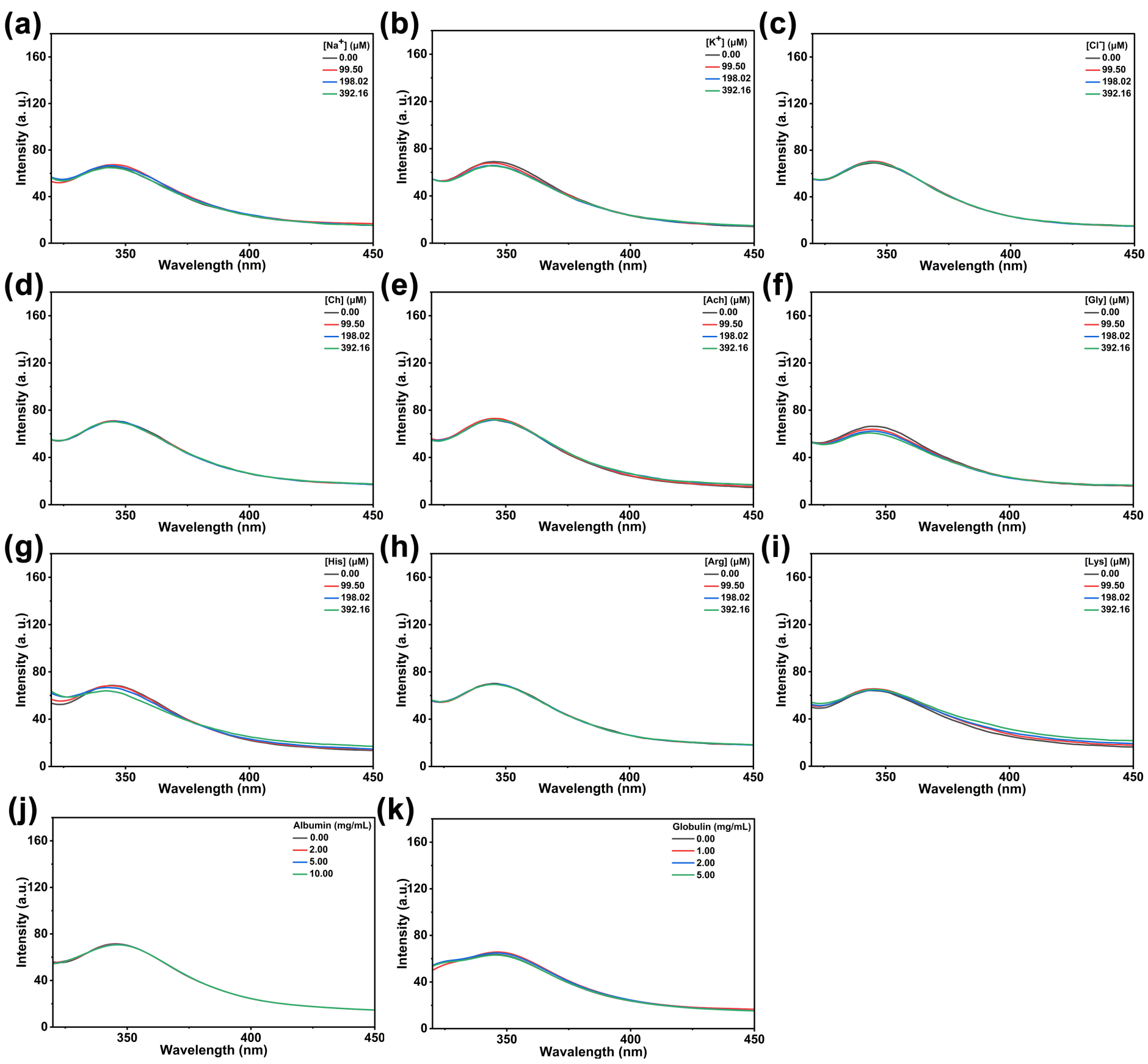

Fig. S7. Competitive fluorescence titration of (a) Na^+, (b) K^+, (c) Cl^-, (d) choline (Ch), (e) acetyl choline (Ach), (f) glycine (Gly), (g) histidine (His), (h) arginine (Arg), (i) lysine (Lys), (j) albumin and (k) globulin in the presence of DQ (1.0 μM) /P6AS (1.0 μM) in aqueous solution, λ_{ex} = 275 nm. The recovery of fluorescence is too small relative to the range of quenching, so no reasonable association constant can be obtained.

3.7 Biosafety evaluation of P6AS.

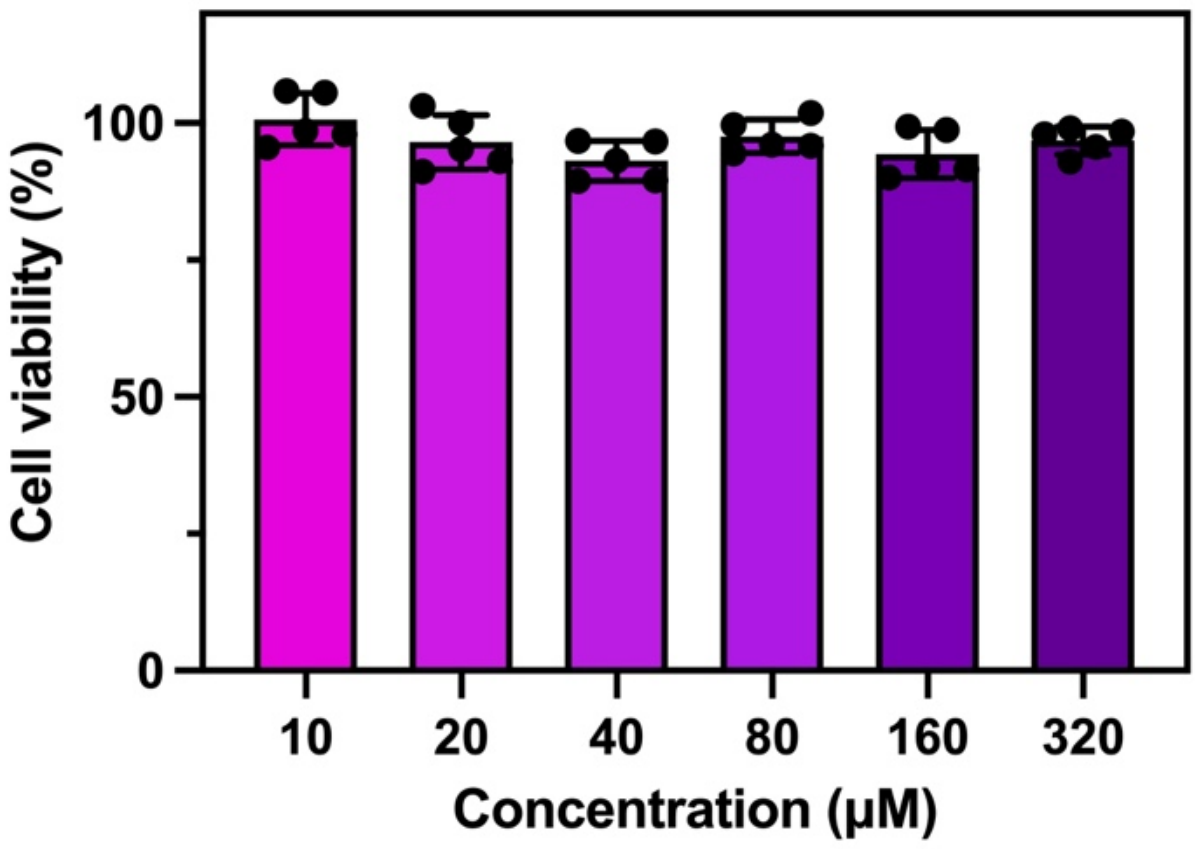

Fig. S8. Viability of HepG2 cells treated with different concentrations of P6AS for 24 h.

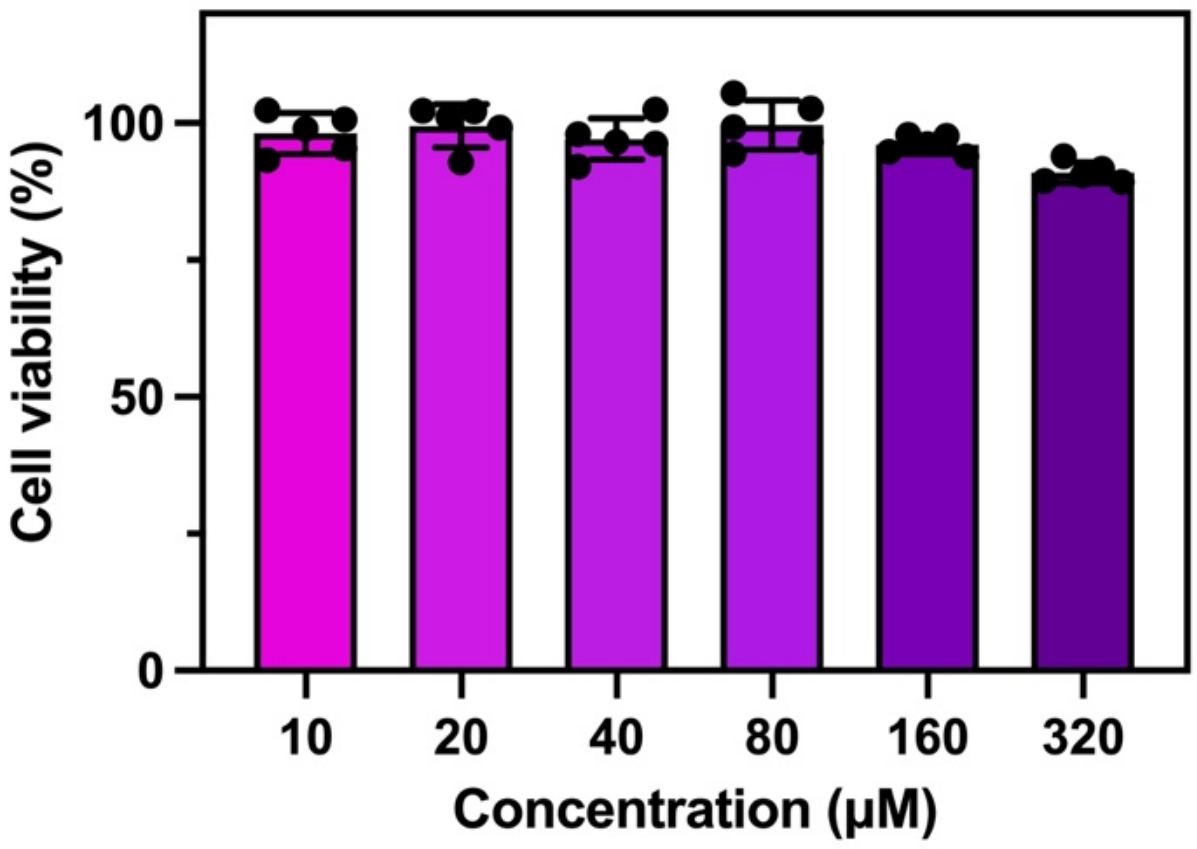

Fig. S9. Viability of 293T cells treated with different concentrations of P6AS for 24 h.

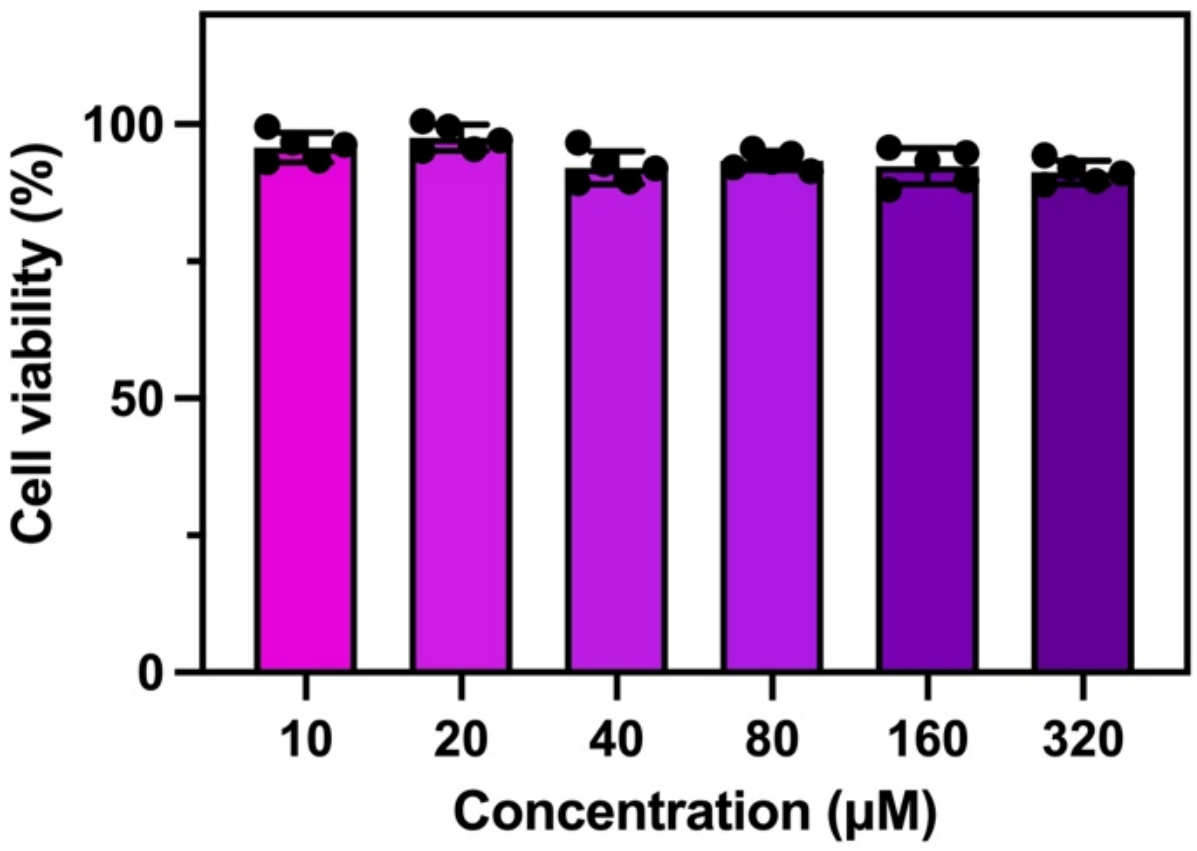

Fig. S10. Viability of A549 cells treated with different concentrations of P6AS for 24 h.

3.8 The storage stability of the EGT/P6AS complex.

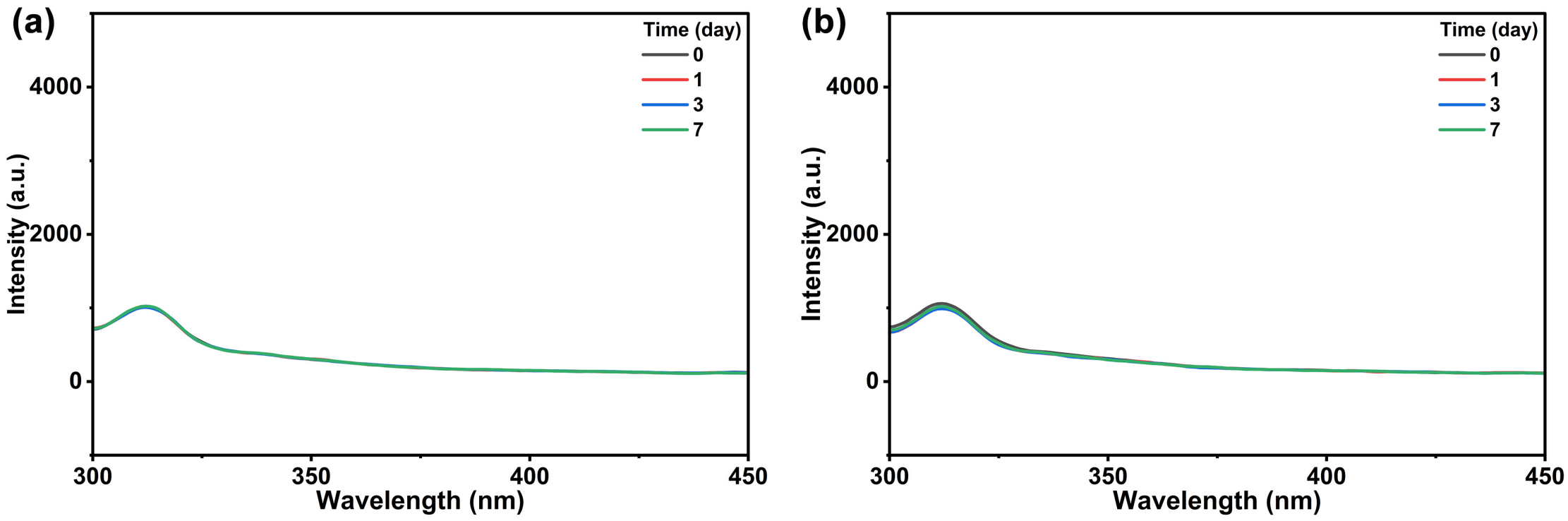

Fig. S11. Fluorescence spectra of EGT (10.0 μM) /P6AS (1.0 μM) after storage at (a) 4 °C and (b) 25 °C. Spectra were acquired after 1, 3, and 7 days of storage, λ_{ex} = 290 nm and λ_{em} = 310 nm.

3.9 Treatment of DQ poisoning by EGT/P6AS *in vitro*.

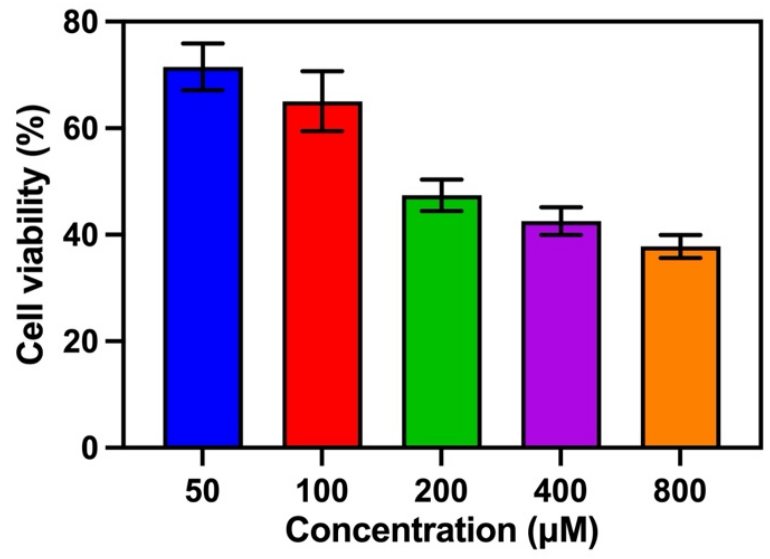

Fig. S12. Viability of HepG2 cells treated with different concentrations of DQ for 24 h.

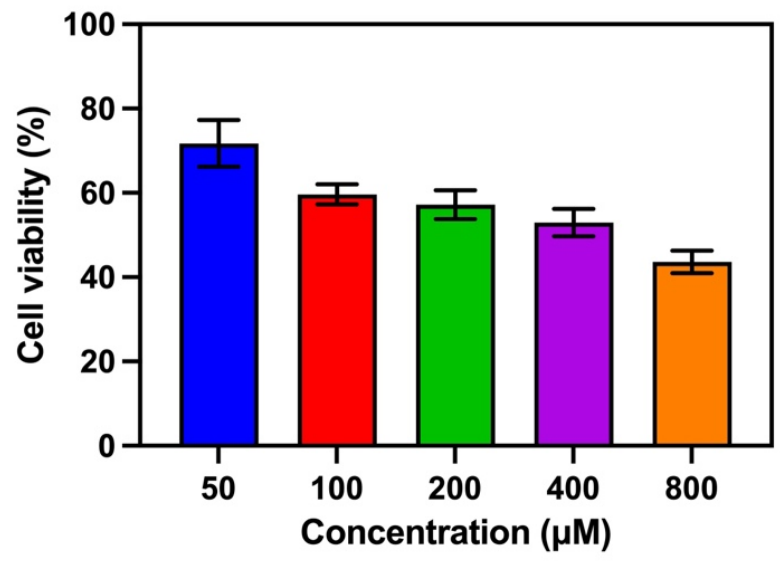

Fig. S13. Viability of 293T cells treated with different concentrations of DQ for 24 h.

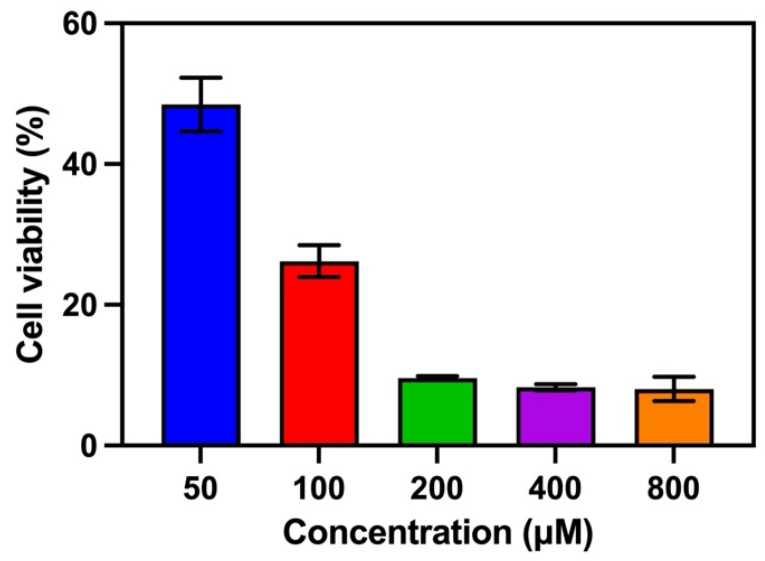

Fig. S14. Viability of A549 cells treated with different concentrations of DQ for 24 h.

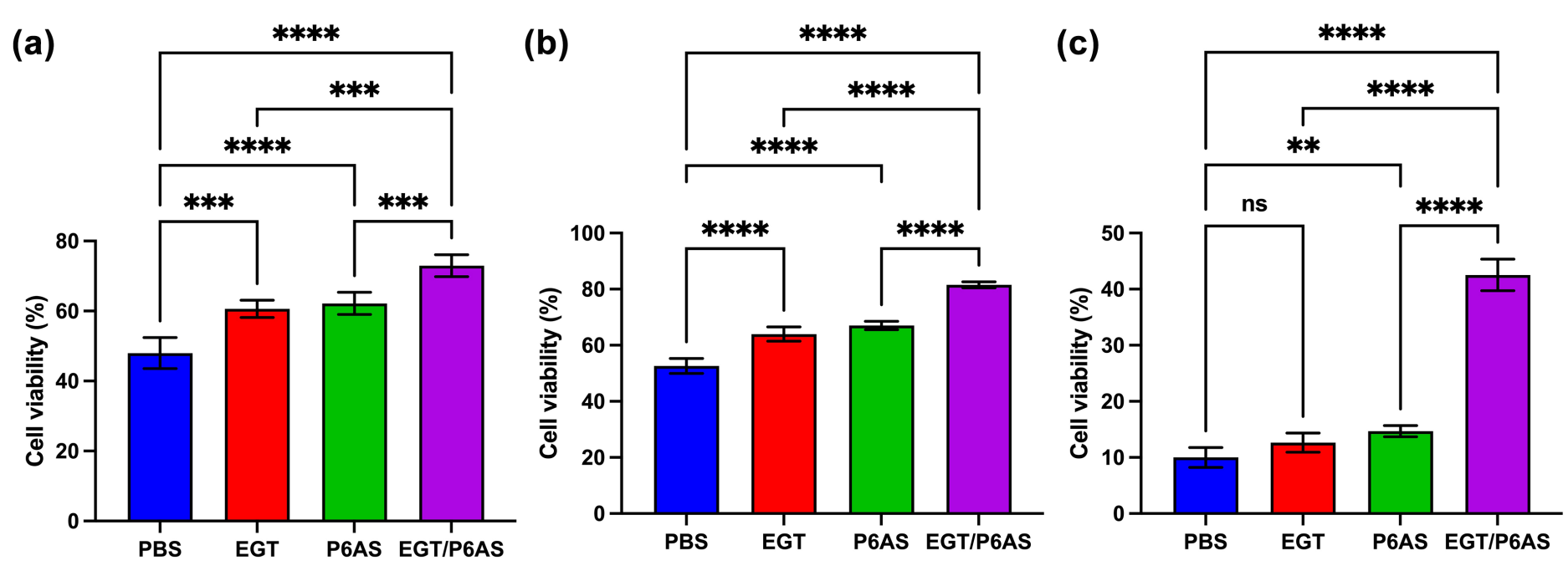

Fig. S15. *In vitro* detoxification efficacy of different formulations against DQ poisoning. Cell viability of (a) HepG2, (b) 293T, and (c) A549 cells after 24 h treatment with DQ (200 μM) in the presence of PBS, EGT, P6AS, or EGT/P6AS. Data are presented as mean ± SD (n = 5). P-values are determined using one-way analysis of variance (ANOVA) test. ns, no significance. $^{**}P < 0.01$, $^{***}P < 0.001$, and $^{****}P < 0.0001$

3.10 Intracellular ROS levels in 293T cells.

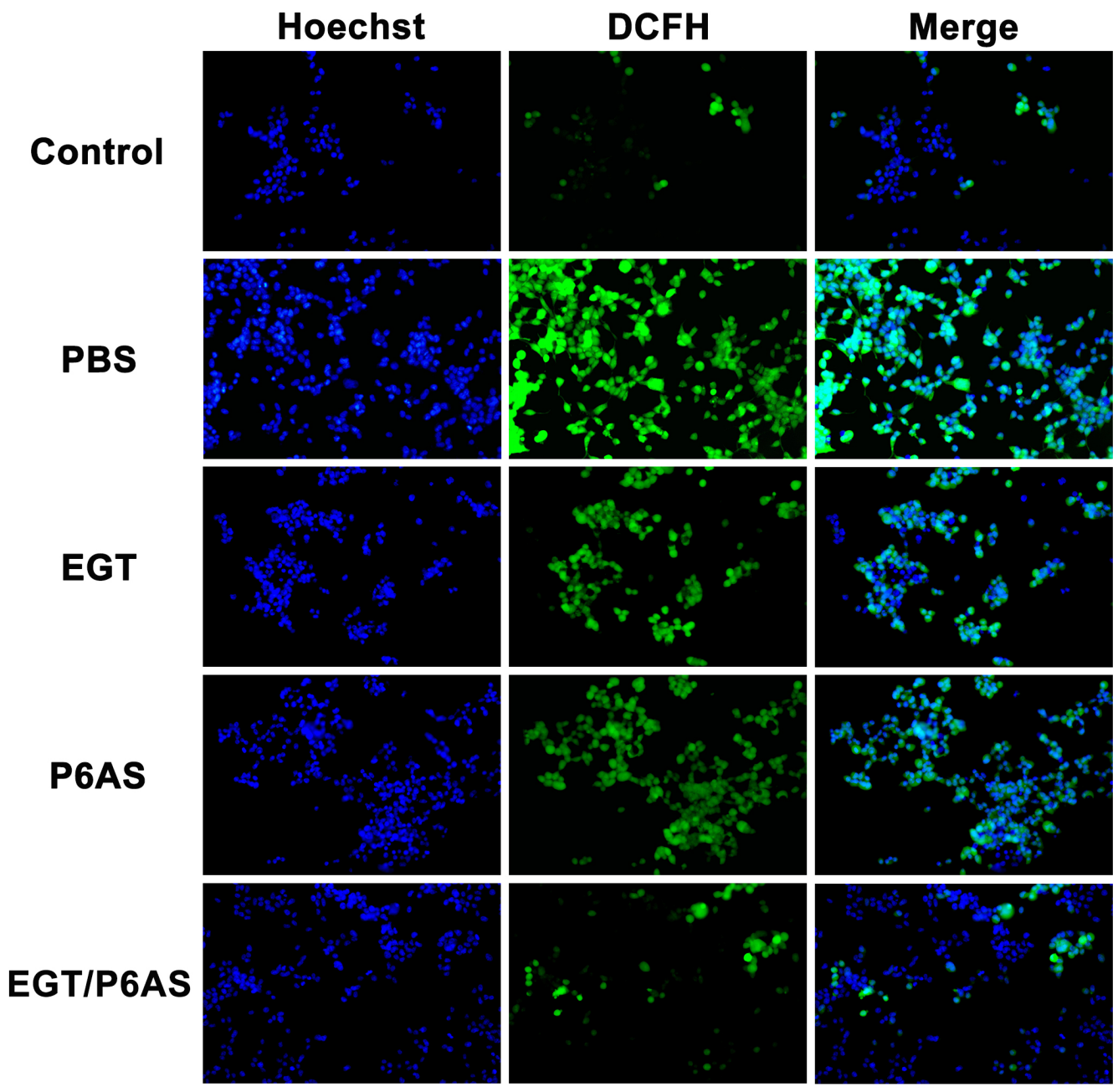

Fig. S16. Representative fluorescence images of 293T cells showing intracellular ROS levels after corresponding treatments (scale bar: 100 μm).

References

1. W. Xue, P. Y. Zavalij and L. Isaacs, *Angew Chem. Int. Ed.*, 2020, **59**, 13313-13319.
2. D. King, C. R. Wilson, L. Herron, C-L. Deng, S. Mehdi, P. Tiwary, F. Hof and L. Isaacs, *Org. Biomol. Chem.,* 2022, **20**, 7429-7438.

3 R. N. Dsouza, U. Pischell, and W. M. Nau, *Chem. Rev.*, 2011, **111**, 7941-7980.

4 J. Gao, J. Li, W. Geng, F. Chen, X. Duan, Z. Zheng, D. Ding and D.S. Guo, *J. Am. Chem. Soc.,* 2018, **140**, 4945-4953.

5 J. H. Tian, S. Huang, Z. H. Wang, J. J. Li, X. Song, Z. T. Jiang, B. S. Shi, Y. Y. Zhao, H. Y. Zhang, K. R. Wang, X. Y. Hu, X. Zhang and D. S. Guo, *Nat. Commun.,* 2025, **16**, 1016.
